# Will the PRESERFLO™ MicroShunt impact the future of trabeculectomy practice? A UK and Éire Glaucoma Society National Survey

**DOI:** 10.1038/s41433-022-02326-6

**Published:** 2022-12-08

**Authors:** Mong-Loon Kuet, Augusto Azuara-Blanco, Keith Barton, Anthony J. King

**Affiliations:** 1 Department of Ophthalmology, Nottingham University Hospitals NHS Trust, Nottingham, UK; 2 Centre for Public Health, School of Medicine, Dentistry and Biomedical Sciences, Queen’s Hospital Belfast, Belfast, UK; 3 Moorfields Eye Hospital NHS Foundation Trust, London, UK; 4 UCL Institute of Ophthalmology, London, UK

**Keywords:** Glaucoma, Implants

## Abstract

**Background/objectives:**

To explore the attitudes of UK glaucoma specialists regarding the current and future practice of trabeculectomy and the novel PRESERFLO™ MicroShunt (PF-MS) device, and intentions to adopt the PF-MS into routine glaucoma surgical practice.

**Methods:**

Online survey of UK and Éire Glaucoma Society members.

**Results:**

43 glaucoma consultants completed the survey. All surgeons performed trabeculectomies (median of 40 annually) and 51% undertook PF-MS procedures (median of 22.5 annually). The mean duration of surgery was reported as 48.9 (SD 13.3) and 31.2 (SD 9.9) min for trabeculectomy and PF-MS respectively (*p* < 0.0001). For surgeons not currently using the PF-MS, 65% planned to do so. Respondents judged completion of 35 trabeculectomies and 10 PF-MS were required to achieve basic competence. 91% of participants predicted their trabeculectomy volume would decrease and 73% expected PF-MS usage to increase. Respondents reported a median of six and four follow-ups within 3 months post surgery for trabeculectomy and PF-MS respectively (*p* < 0.0001). Respondents reported trabeculectomy required more post-operative interventions than the PF-MS and 81.8% judged the patient experience to be better with the PF-MS. The PF-MS was deemed suitable for early visual field loss by 72% of respondents, severe visual field loss by 35% and normal tension glaucoma by 21%.

**Conclusion:**

The PF-MS has seen rapid adoption in the UK. Respondents predict its usage will significantly increase whilst trabeculectomies will decrease. They report the PF-MS is quicker to learn and perform, and requires less post-operative follow-ups and interventions which may facilitate a more efficient service delivery for patients requiring glaucoma surgery.

## Introduction

Introduced in 1968 [1], trabeculectomy is the most performed glaucoma surgical intervention worldwide. It is an incisional surgery intervention recommended by the National Institute of Health Care and Excellence (NICE) [2], the European Glaucoma Society [3] and the American Academy of Ophthalmology [4]. Trabeculectomy has an established evidence base supporting its effectiveness [5–7]. However, trabeculectomy is an invasive procedure with potential sight-threatening risks and is a complex intervention requiring high-level training and significant post-operative management [8, 9]. Surgical innovations including minimally invasive glaucoma surgery and ab externo bleb forming procedures have gained traction as treatment options that may provide safer and less traumatic alternatives to conventional incisional surgery with a faster recovery time. Such procedures differ from trabeculectomy as there is less disruption to the conjunctiva and sclera. There are indications that the COVID-19 pandemic has facilitated changes to glaucoma surgical practice in the UK. Alternative procedures that are faster to perform and require less follow-up are being considered favourably, resulting in less trabeculectomies and an increase in alternative options [10]. The PRESERFLO™ MicroShunt (PF-MS) (Santen, Osaka, Japan) is a relatively new subconjunctival ab externo bleb forming device that received CE-marking in 2012 but has only been available for use in Europe and the UK since early 2019. The MicroShunt is 8.5 mm long with a 70 µm diameter lumen, manufactured from a biocompatible polymer known as poly(**s**tyrene-**b**lock-**i**sobutylene-**b**lock-**s**tyrene) ‘SIBS’ [11]. The device is implanted subconjunctivally mimicking the drainage mechanism of a trabeculectomy. The procedure is augmented with mitomycin C (MMC). Long-term data on outcomes are limited but initial clinical studies have shown substantial reductions in IOP can be achieved with a favourable safety profile [12–17]. There is therefore interest in whether the PF-MS has the potential to be offered as an alternative to trabeculectomy.

As the UK emerges from the COVID-19 pandemic it is important to recognise sustained future trends in the use of the PF-MS and how the device may impact glaucoma surgical trabeculectomy practice and volume. Such information would be of interest to glaucoma specialists, clinical leaders, healthcare commissioners and national bodies overseeing and designing glaucoma services. Any changes in surgical practice will also have implications for the availability of trabeculectomy cases for training. Despite the increasing uptake of the PF-MS in the UK [10], little is known regarding the attitudes of glaucoma specialists as to which patients may benefit from the PF-MS, their perceptions of the usage of the PF-MS in the future, and the relative importance of the perceived advantages of the device, in terms of recovery time and learning curve. Such insight will help clarify how the PF-MS fits in the treatment algorithm for glaucoma and inform the design of potential future randomised controlled trials exploring the effectiveness of the device.

## Methods

An online survey was emailed to glaucoma consultants on the United Kingdom and Éire Glaucoma Society (UKEGS) contact list on 17th November 2021. An email reminder was sent after 6 weeks to capture any non-responders. UKEGS is the professional body representing glaucoma specialists in the UK and the Republic of Ireland. In total 26 questions were asked, covering five domains. The questions were designed to establish attitudes to: current practice (eight questions) for the PF-MS and trabeculectomy surgery; predicted trends in surgical practice (ten questions); post-operative care for both procedures (four questions); patient suitability for the PF-MS (one question); and the design of future studies comparing both procedures (three questions). Anonymous survey responses were viewed using Microsoft Excel (version 14.3.1). Statistical analysis was undertaken using GraphPad Prism (version 9.3.1). The survey used is available as Supplementary material (Appendix 1).

Recent changes in the UKEGS membership registration system mean that UKEGS does not currently record the designation of its members, so the exact number of glaucoma consultants surveyed is unknown. However, two recent publications [18, 19] have indicated the numbers to be 69 and 72; we have chosen the larger number of 72 to estimate our response rate on.

## Results

A total of 43 UK glaucoma consultants completed the survey, providing an estimated survey response rate of 59.7%. All participants performed augmented trabeculectomy and the median number performed annually was 40; 51.2% of specialists also performed the PF-MS procedure with a median number undertaken over the preceding year of 22.5. Respondents deemed a median of 35 trabeculectomies was necessary to achieve basic competency for this procedure, whilst for the PF-MS the median number was ten. The mean self-reported duration for a trabeculectomy was 48.9 (SD 13.3) min; for specialists using the PF-MS this was 31.2 (SD 9.9) min (*p* < 0.0001, unpaired *t*-test) (Table 1).

**Table 1:** Respondents’ current surgical practice patterns.

Question on current surgical practice	Response
*Trabeculectomy surgery*	
Median number performed per year	40 (range 10–100)
Mean duration	48.9 mins (range 30–90 min)
Median number for basic proficiency	35 (range 10–100)
Number of follow-ups within 3 months	6 (range 3–10)
*PRESERFLO™ MicroShunt surgery*	
Median number performed per year	22.5 (range 3–80)
Mean duration	31.2 mins (range 17–50 min)
Median number for basic proficiency	10 (range 5–50)
Number of follow-ups within 3 months	4 (range 3–8)

Ninety percent of specialists performing both procedures anticipated their trabeculectomy volume would reduce; a median reduction of 50% was predicted (range 20–90%). In contrast 72.7% of respondents currently using the PF-MS anticipated they would perform more of this procedure; a median increase of 30% was predicted (range 10–100%). Respondents' free text reasons for the increase are summarised in Table 2. Of the respondents not currently using the PF-MS, 61.9% planned to do so in the future. A minority (39.5%) of participants cited the cost of the PF-MS as a factor for not currently undertaking the procedure, however, 72.1% of all respondents believe the PF-MS will play an important role in addressing the COVID-19 pandemic backlog.

**Table 2:** Reasons given for respondents’ predicted increase in PRESERFLO™ MicroShunt surgery.

Reasons for performing PRESERFLO™ MicroShunt surgery	Number of responses (% of specialists performing both procedures)
Reduced post-operative follow-ups	11 (50.0)
Shorter surgical duration	9 (40.9)
Less complications	7 (31.8)
Good IOP lowering efficacy	7 (31.8)
Suitable for earlier stage disease	5 (22.7)
Better predictability of IOP outcomes	3 (13.6)
Suitable for patients with multiple co-morbidities	3 (13.6)
Patient preference	1 (4.5)
Suitable for failed trabeculectomy cases	1 (4.5)
Better visual recovery	1 (4.5)

Respondent’s free-text responses are summarised in this table.

Of the specialists that performed both procedures, 77.3% reported that trabeculectomy required more post-operative interventions (including bleb needlings, massage, suture removal and antimetabolite injections) than the PF-MS, 18.2% reported both procedures required the same number of post-operative interventions whilst 4.5% experienced more interventions with the PF-MS than a trabeculectomy. The majority of respondents (81.8%) performing both procedures believed the post-operative patient experience was better with the PF-MS. The remaining respondents (18.8%) observed no difference in patient experience between the two procedures.

**Fig. 1:**
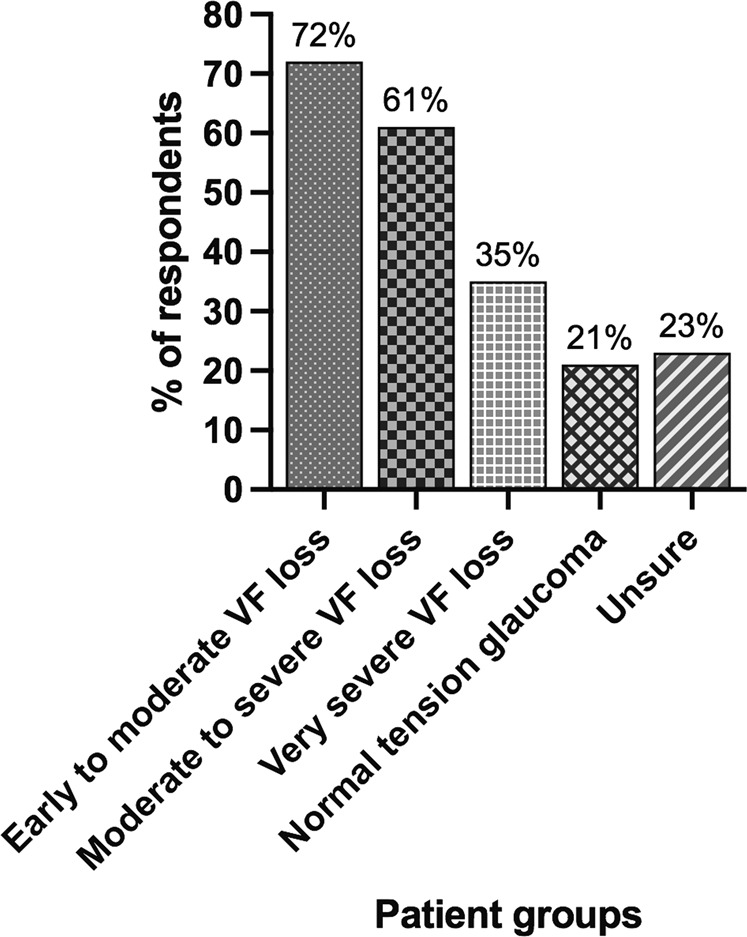
Respondents’ perceptions on suitable patient groups for the PRESERFLO™ MicroShunt. Respondents were able to choose more than one patient group. VF visual field. *n* = 43.

Seventy-two percent of respondents believed PF-MS was a suitable intervention for mild-moderate visual field loss, but fewer than half of these (35%) believed it was suitable for advanced visual field loss or normal tension glaucoma (21%) (Fig. 1).

**Fig. 2:**
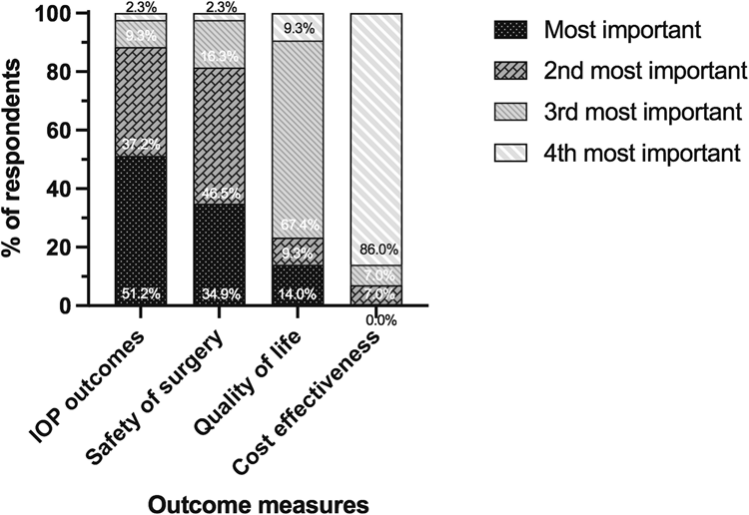
Respondents’ perceptions on the most important outcome measure to evaluate in a future RCT on the PRESERFLO™ MicroShunt versus trabeculectomy. Participants were asked to rank the four outcome measures in order of importance from first choice to last choice. *n* = 43.

**Fig. 3:**
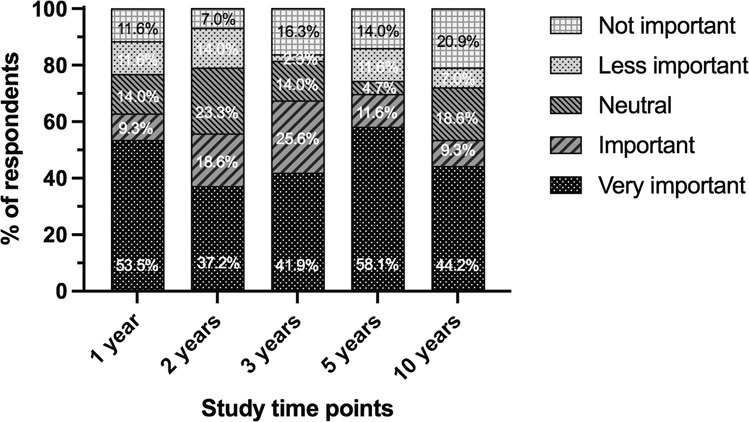
Respondents’ perceptions on the importance of comparing PRESERFLO™ MicroShunt versus trabeculectomy outcomes at 1, 2, 3, 5 and 10 years in a future RCT. Participants were asked to state the importance of comparing outcomes at these time points on a scale of 1–5. *n* = 43.

Ninety-five percent of respondents stated they would be willing to randomise patients in a randomised controlled trial comparing the two procedures if one was undertaken. Intraocular pressure (IOP) was ranked the most important outcome measure to evaluate by the highest proportion of participants (51.2%) (Fig. 2). A clinically significant difference in IOP when comparing the two procedures was most commonly defined as >3 mmHg and 2–3 mmHg (48.8% and 44.2% of respondents respectively), followed by 1–2 mmHg (7%). Measurement of outcomes at 3 and 5 years was judged to be the most important time for reporting clinical trial results (Fig. 3).

## Discussion

In the recent past, novel glaucoma surgical interventions have been introduced in clinical practice. Although the literature on the PF-MS is currently limited to short-term follow-up, small case numbers and mostly observational studies, these initial studies have shown encouraging results on IOP lowering efficacy and safety [12–15, 20]. To date, there is only one randomised control trial (RCT) comparing trabeculectomy with the PF-MS [16]. The 1-year interim results showed a mean IOP reduction from 21.1 mmHg to 14.3 mmHg with a mean of 0.6 post-operative glaucoma medications in the PF-MS group, and from 21.1 mmHg to 11.1 mmHg with a mean of 0.3 post-operative medications in the trabeculectomy group. Fewer post-operative interventions were required for the PF-MS (40.8% of patients) compared to trabeculectomy (67.4%) by year one. The safety profile was acceptable in both groups with the incidence of hypotony higher for trabeculectomy at 49.6% compared to 28.9% for the PF-MS. Vision-threatening complications were similar for both groups, occurring in 1.0% and 0.8% of the PF-MS and trabeculectomy patients, respectively.

The widespread use of trabeculectomy in this survey is consistent with existing studies on glaucoma surgical practices in the UK [10, 21] and its position as the most established surgical treatment for glaucoma in the UK. In contrast, just over half of respondents currently also performed the PF-MS, and the median annual number, performed from November 2020 to November 2021, was 22.5 procedures. Considering PF-MS has only been commercially available in Europe since early 2019, this indicates a rapid adoption by a large proportion of specialists during a period that coincided with the second and third waves of the COVID-19 pandemic. National guidance [22] on glaucoma surgery at this time recommended that interventions with reduced contact time and fewer attendances should be considered which is likely to have accelerated adoption of the PF-MS.

Just less than two-thirds of the respondents not currently using the PF-MS, planned on adopting it in the future. The majority of respondents stated their trabeculectomy volume would reduce in the future by a predicted average of 50%, and their PF-MS volume would increase by a predicted average of 30%. The most common reasons cited for use of the PF-MS were the need for less post-operative visits (50% of surgeons), shorter surgical duration (40.9%), the perception that the PF-MS had good IOP lowering efficacy (31.8%) and was safer than trabeculectomy (31.8%). Although recent studies have already established that trabeculectomy numbers reduced during the COVID-19 pandemic and alternative glaucoma procedures increased [10, 23], these findings indicate the trend will be sustained long-term with a significant shift towards the PF-MS. However, there is no evidence at present to indicate the long-term effectiveness of the PF-MS and current evidence suggests in the short term that it is not as effective as trabeculectomy at lowering IOP [16]. PF-MS adoption is also likely to be further facilitated by the significant differences in the learning curve and surgical duration between the two procedures highlighted in this survey, making adoption easier. The median duration for performing a trabeculectomy (48.9 min) was 50% longer than for the PF-MS (31.2 min), in keeping with the fewer surgical steps required to perform the PF-MS [24] and making training easier for the PF-MS.

The most notable benefit of such a change is a potential reduction in the extensive surgical backlog of glaucoma patients, a view shared by the majority (72.1%) of respondents. However, any reduction in trabeculectomy surgery is likely to have opportunity costs for training, reducing opportunities for learning trabeculectomy in the future and subsequent surgical versatility as consultants [25–27]. This is especially pertinent as the only RCT undertaken so far indicates the IOP reduction achieved with the PF-MS is considerably less than that of trabeculectomy [16] and no long-term RCT data exists. Wholesale conversion to the PF-MS risks losing the trabeculectomy skills required to achieve the low IOP outcomes required for normal tension glaucoma and advanced glaucoma [7].

The survey’s finding that the PF-MS required an average of two less outpatient visits than trabeculectomy during the three-month post-operative period (*p* < 0.0001), and required less post-operative interventions is again relevant to the current glaucoma capacity issues [28]. This may also explain the majority of surgeons’ (81.8%) belief that the PF-MS provides a better patient experience compared to trabeculectomy.

There is limited literature available on the IOP outcomes from the PF-MS, which suggest a range of 11.4–14.5 mmHg achieved at 12 months [12–16, 24]. On the basis of this limited evidence and their own personal experience, the majority of respondents (72.1%) support the use of the PF-MS in primary open-angle glaucoma (POAG) patients with early to moderate visual field loss. This is understandable as many of those with less severe visual field loss may not require very low IOPs to prevent further visual field loss. However, a large proportion of respondents (60.5%) also deemed POAG patients with moderate to severe visual field loss suitable for treatment and 34.9% felt it was a suitable intervention for severe glaucoma. This is more controversial as these patients are likely to require low IOPs and the evidence that currently exists suggests that the PF-MS does not achieve as low an IOP as trabeculectomy and therefore may not be as effective in these patients [16]. A minority of surgeons (20%) believed the PF-MS was a suitable option for normal tension glaucoma (NTG) which may be reflective of the few studies which only included low numbers of this glaucoma subtype for evaluation [14, 20, 24, 29]. The low IOPs needed to prevent further visual field loss in advanced glaucoma and NTG may not be achieved with the PF-MS procedure and extensive adoption of the PF-MS may have consequences for the successful surgical management and vision preservation of these patients in the future.

Further evidence comparing trabeculectomy and the PF-MS would greatly enhance our understanding of the position of these interventions within the spectrum of glaucoma surgical treatments. Understanding outcomes that would be considered important in future studies of the PF-MS would inform future trial design. IOP outcomes and safety of surgery were prioritised as the two most important outcome measures to evaluate in future studies. For IOP a clinically important difference between trabeculectomy and the PF-MS was considered as 2–3 mmHg and >3 mmHg (44.2% and 48.8% of respondents, respectively). This information will be useful to inform the design of potential future RCTs comparing these two procedures, and this survey has confirmed there is widespread interest from UK surgeons to participate in such a study.

The limitations of this study are acknowledged. There were a modest number of responses owing predominantly to the respondents coming from a relatively limited pool of UK glaucoma specialists. The exact number of glaucoma consultants on the UKEGS survey list is unknown and so our response rate is an estimate based on recently published studies using the same survey pool [18, 19]. Questions pertaining to the number of procedures performed and duration of surgery relied on retrospective self-reporting and therefore were prone to recollection bias. This survey specifically focussed on future patterns of practice for the PF-MS and trabeculectomy, and so it is does not give an insight into alternative glaucoma procedures that could also influence trabeculectomy practice.

In conclusion, the PF-MS has seen widespread adoption since its introduction to the UK only four years ago. This trend is likely to be sustained beyond the COVID-19 pandemic, whilst the volume of trabeculectomy is predicted to decrease significantly. While the PF-MS may have a role in tackling the surgical backlog of patients, there is limited long-term outcome data. Further comparative studies are needed to determine its long-term efficacy and to clarify which patients will benefit from this procedure.

Supplementary information are available at nature.com/eye.

### Summary

#### What was known before

- The PRESERFLO MicroShunt is a novel ab externo bleb forming device introduced in the UK in 2019.
- Initial studies have shown substantial IOP reductions with a favourable safety profile, but long term data on its effectiveness is awaited.
- There is interest in whether the PRESERFLO MicroShunt can be offered as a less invasive alternative to trabeculectomy.

#### What this study adds

- The PRESERFLO MicroShunt has seen rapid adoption in the UK, with half of the study’s respondents using this device despite the lack of long term data on its effectiveness.
- They predict its usage will significantly increase whilst trabeculectomies will decrease.
- Glaucoma consultants report the device is quicker to learn and perform, and requires less post-operative follow-ups and interventions than a trabeculectomy, which may facilitate a more efficient service delivery for patients requiring surgery.

## Supplementary information

Appendix 1

## Data Availability

All data generated or analysed during this study are included in this published article (and its supplementary information files).
