# Supplementary material for: Will the PRESERFLO™ MicroShunt impact the future of trabeculectomy practice? A UK and Éire Glaucoma Society National Survey
Source: Eye (Lond). 2022 Dec 8;37(11):2252–6. doi: 10.1038/s41433-022-02326-6 (PMC9735154; doi:10.1038/s41433-022-02326-6)
Supplement: Supplementary file 1 — Appendix 1 [file 41433_2022_2326_MOESM1_ESM.pdf]

Incisional Glaucoma Surgery Survey

We are grateful for you help with this survey which we hope will provide more information regarding the use of trabeculectomy and Preserflo in the management of glaucoma patients requiring incisional surgery. We realise that not all respondents undertake both trabeculectomy and Preserflo, if this is the case we would be grateful if you would answer those related to the operation you undertake and any others you have an opinion on. Please be aware the questions appear as you go along so do not submit the survey until the last questions (Q27). It will take about 6 minutes to complete the survey.

* Required

1. Do you undertake trabeculectomy? *

◯ Yes

◯ No

2. In a normal year (non-COVID) on average how many trabs do you undertake annually?

3. Do you use or have you used Preserflo? *

◯ Yes

◯ No

4. In the last year how many Preserflos have you undertaken?

5. In your opinion what number of **trabs** do you believe are necessary to complete in order to be considered competent? *

6. In your opinion what number of **Preserflos** do you believe are necessary to complete in order to be considered competent?

7. If you undertake both trab and Preserflo do you anticipate the number of trabs you undertake will reduce now that you are using Preserflo? *

○ Yes

○ No

○ I don't undertake both procedures

8. If yes, what % reduction do you estimate it will decrease?

9. Why?

10. If you undertake both trab and Preserflo do you anticipate the number of Preserflos you undertake will increase in the future? *

- ◯ Yes
- ◯ No
- ◯ I don't undertake both procedures

11. If yes, by what % do you estimate it will increase?

12. Why?

13. In patients on MMT who require incisional glaucoma surgery do you think Preserflo (in general) is suitable in the following situations (please tick all that apply): *

- ☐ Patient with early-moderate visual field loss (MD<-10dB)
- ☐ Patients with moderate-severe visual field loss (MD>-10<-20dB)
- ☐ Patients with very severe visual field loss (MD>-20dB)
- ☐ Patients with normal tension glaucoma
- ☐ Unsure

14. On average how many follow-up visits does a patient under your care require in the 3 months following trabeculectomy?

15. On average how many follow-up visits does a patient under your care require in the 3 months following Preserflo?

16. In your experience do you believe trab or Preserflo require more post-operative interventions (antimetabolite injections / needlings etc)? *

- ○ Trabeculectomy
- ○ Preserflo
- ○ Neither - the same
- ○ I don't undertake both procedures

17. Do you believe the patient experience is better following trab or Preserflo surgery? *

- ○ Trabeculectomy
- ○ Preserflo
- ○ Neither - the same
- ○ I don't undertake both procedures

18. It you undertake trabeculectomy, on average (in minutes) how long does it take you to undertake a trab operation?

19. If you use or have used Preserflo, on average (in minutes) how long does it take you to do a Preserflo operation?

20. Does the fact that it cost £1000 for a Preserflo put you off using it? *

- Yes
- No

21. Comparing Trab and Preserflo – what do you think are the most important characteristics to explore further – please rank importance by using arrows on side (top most important to bottom least important) *

- IOP outcomes
- Safety of Surgery
- Patient Quality of Life
- Cost Effectiveness

22. What difference in IOP outcome do you consider to be a clinically important difference between preserflo and trab? *

- < 1mmHg
- 1-2mmHg
- 2-3mmHg
- > 3mmHg

23. Do you think that Preserflo potentially has an important role in managing the glaucoma surgery back log associated with COVID? *

- ◯ Yes
- ◯ No

24. If you are currently not using Preserflo do you intend to start using it in the future? *

- ◯ Yes
- ◯ No
- ◯ I am currently using Preserflo

25. If you undertake both trabeculectomy and Preserflo would you be willing to randomise patients into a randomised controlled trial comparing the two procedures if one was undertaken? *

- ◯ Yes
- ◯ No
- ◯ I don't undertake both procedures

26. On a scale of 1 -5 (1 being not important)

How important do you think it is to have a comparison of outcomes between trab and preserflo at the following timepoints *

	1	2	3	4	5
1 year	○	○	○	○	○
2 years	○	○	○	○	○
3 years	○	○	○	○	○
5 years	○	○	○	○	○
10 years	○	○	○	○	○

27. We would like to share the results of this survey with respondents – if you would like us to do this please provide your email address below

This content is neither created nor endorsed by Microsoft. The data you submit will be sent to the form owner.

Microsoft Forms
